# Dilemma breaking in quantum games by joint probabilities approach

**DOI:** 10.1038/s41598-022-17072-8

**Published:** 2022-08-05

**Authors:** Alexis R. Legón, Ernesto Medina

**Affiliations:** 1 Laboratorio de Física Estadística de Medios Desordenados, Instituto Venezolano de Investigaciones Científicas (IVIC), Carretera Panamericana, Km 11, Altos de Pipe, 1020A, Caracas, Venezuela; 2 Universidad Técnica Federico Santa María, Av. España, 1680 Valparaíso, Chile; 3 Departamento de Física, Colegio de Ciencias e Ingeniería, Universidad San Francisco de Quito, Diego de Robles y Vía Interoceánica, Quito, 170901 Ecuador

**Keywords:** Information technology, Information theory and computation, Statistical physics, thermodynamics and nonlinear dynamics

## Abstract

Classical games get fundamentally modified in the quantum realm because of non-locality and entanglement, that bypass some of the crucial features of the classical problem that define a *dilemma*. We will analyze how the dilemma can be shunted and even completely eliminated by the players using quantum strategies from the viewpoint of joint probabilities. In this approach, the game information (entropy) needs to be incorporated into the game strategies. We also connect the potential of the formalism of quantum games with the transmission of quantum information in quantum noisy channels and recent considerations of the connection between thermalization mechanisms in statistical mechanics, the many body problem and cooperative games considered here in the quantum regime.

## Introduction

It is by now understood that information is fundamentally rooted in physics^1,2^. As ultimately physics is quantum, so is information. Some crucial barriers of classical information theory have been circumvented by its quantum counterpart opening the field of quantum computation to new horizons mainly due to the availability of entanglement as a fundamental resource^1,2^. A place where information plays a central role is in the branch of mathematics of Game Theory, which provides tools for analyzing situations of conflict in which parties, called players, make decisions that are interdependent. Therefore, each player considers the other player’s possible decisions or strategies, to formulate their best strategy. However, the optimal strategies of the players describe a solution to the game when the conflict situation is resolved. Otherwise, we obtain dilemmas, situations in which there is no optimal solution to the game.

Although Game theory was originally developed in the mathematical context, attempting to describe games of chance, and gambling, it quickly gained footing as a foundation for microeconomics. The first development where the so called sum zero games where players are at completely at odds and had no reason to cooperate. Later the restriction was lifted and the field of cooperative Game Theory was born. The quantum extension of classical cooperative games is known as Quantum Games^2–9^(QG). The quantum extension of games has been recently reviewed for its possible role in economics as a new environment for quantum technology and negotiations^10–12^.

In the Quantum Games scheme of Eisert^6,7^, players’ strategies are particular local unitary transformations performed on an initial maximally entangled state in a bipartite Hilbert space. After the players strategies are in place, the quantum state passes through a disentangling gate that yields the final state. This state is subsequently measured for four ’quantum’ probabilities (henceforth just probabilities). The pay-off relations of the game are expressed in terms of the pay-off entries of the corresponding bimatrix and the resulting probabilities.

A feature of QG is that entanglement interferes with the dilemma present in the classical games^6,7^. Classically, this dilemma consists in that no player can win without lowering another player’s expected pay-off. In this sense, for QG, one can say that the dilemma of the original game can completely disappear, i.e, one says *the dilemma of the game is broken*. Some constraints of the classical games are lifted in their quantum counterpart, thus interfering with the dilemma, which opens the possibility to obtain an equilibrium where both players win with an acceptable pay-off within the possible pay-offs for the strategies available in the game. Incorporating entanglement to the initial state of the game then generates strategies that are not initially available to the players^7^. A mathematical formulation of the strategies allow exploring the competitive interactions between quantum and classical players^5,13^.These strategies are tested here for the Prisoner’s Dilemma^6,7^ and the Battle of the Sexes^14^. Likewise, this approach can also be evaluated for other games, such as the Chicken Game^7^. Also, these competitive interactions are observed in quantum multiplayer games^15–18^ and interactions in quantum networks^19^, among others.

The description of QG in terms of joint probabilities was first introduced by Iqbal and Cheon^20^, who start from the Einstein-Podolsky-Rosen (EPR) configuration^13^ that manisfestly depends on an entanglement measure. Furthermore, the approach introduces the notion of symmetry of QG^21^ in probabilistic terms. Quantum games are then characterized by the nature of its joint probabilities; if these are factorizable, it is a classical game, otherwise, it is a QG^21,22^.

Information in QG has been developed in the references^23–25^. In the latter work, reference is made to the density operator of quantum states when determining the capacity of the game. In our approach, the information capacity of QG will be a function of the probabilities of the players in the frame of Non-factorizable probabilities for the players. In Ref.^23^, the Nash Equilibrium for games with mixed strategies was obtained when the entropy is minimal. The main result of our work is to put forth a formulation where we approach QG from the joint probabilities description.

For quantum information theory, a quantum channel is a positive definite, trace-preserving linear map between spaces of operators^1^. The capacities of quantum channels, including quantum channels with noise, have attracted much attention in quantum communication^1,26^. Intuitively, the information capacity of a channel is the maximum amount of information that can pass through a channel^1,27^. This interest was stimulated by the interplay between quantum communication theory, quantum information ideas and quantum computing. Unlike classical channels, which are adequately characterized by a single capacity, quantum channels have several distinct capacities^26^. Below we will discuss how QG can be related to quantum channels and illustrate how this connection bears on quantum communication and the dissolution of the game dilemma.

Quantifying entanglement can be assessed through the tools developed in references^28–30^. Here, we propose to write the parameters $\epsilon _{i}$, proposed by^22^, corresponding to the scheme of joint probabilities and at the same time to determine the inequalities for the Nash equilibrium. In addition we elaborate on the applications of this formulation in connection to the games to determine the equilibria in the QG that *break dilemmas*.

Two very recent additions to the literature for the applicability of game theory to physical systems is the analysis of the open question of thermalization processes to reach equilibrium^31^. In this work, they establish connection between concepts of game theory and statistical mechanics where, e.g. the payoff play the role of the entropy or negative energy, defection point analogous to initial state. They contemplate the cooperation between players (probability of states) in the classical game that defines a Nash equilibrium, and propose it as a possible route to thermalization. Here we broaden the question to the possibilities for quantum evolution.

On the other hand, from the focus of pure game theory, a recent paper^32^ discussed the relative efficiencies of classical and quantum games with quantum mixed Pauli strategies, and found that they are closer to the Pareto optimal than their classical counterparts. Here we report, in that direction, of further possibilities of quantum strategies that allow for a large gap in efficiency with their classical counterparts.

This work is organized as follows: In “Definitions for game theory” section, we introduce important definitions in game theory^3,4,33,34^. In “Traditional approach in QG”, we show the traditional approach to QG. In “Non-factorizable probabilities for quantum games”, we show the relevant aspects of the formalism proposed by^22^, which we use in “New approach to QG” section, in order to connect the joint probabilities approach to QG^6,7^. In Sect. 5, we propose the Joint Probabilities Approach to QG, which is the main contribution of this work. We show the advantage of the joint probabilities approach versus the traditional quantum formalism are: (a) The ease and clarity with which equilibrium situations can be derived in the probabilistic approach (b) The avoidance of non-local treatment of quantum strategies (c) addressing quantum games in terms of the dissolution of the dilemma which is the centerpiece problem in cooperative games. We also show through the formalism, that the breaking of the dilemma can always be achieved by the appropriate local strategies. Furthermore, in section “Information capacity of a quantum game from the joint probabilities”, we propose a way to measure the information capacity in QG from the joint probabilities and show that the games, understood as quantum channels, present better performance than the usual quantum channels, which allows for improved maximization of information transfer. Finally we apply our formalism to two games in section “Joint probabilities approach and information in QG”. We end with a summary and conclusions.

## Definitions for Game Theory

Game theory is a branch of mathematics that is used in modelling situations in which individuals with conflicting interests interact and can cooperate, such that the results depend on the actions of all the participants^3,4,33,34^. The participants in a game strive to maximize their utilities by choosing particular courses of action. Because the actions of other players matter, a player’s utility depends on the profile of the actions chosen by all the participants. In this way, for a game, the following definitions are crucial:

**Players**: *They are the participants who make strategic decisions within the context of the game. Each player’s goal is to maximize its utility by a choice of actions or moves.*

**Move**: *An action or move by player* *i*, *denoted *$a_i$,*is a choice he can make.*

*The i-th player move set, *$A_i = \lbrace a_i \rbrace$, *is the entire set of actions available to him. An action combination is an ordered set* $a = \lbrace a_i \rbrace , (i = 1, . . . , n)$ *of one action for each of the n players in the game.*

**Strategy**: *The i-th player (pure) strategy* $s_i$ *is a rule (or function) that associates a player’s move with the information available at each instant of the game. A player’s mixed strategy is a probability measure on the player’s space of pure strategies.*

*Player i strategy set or strategy space *$S_i = \lbrace s_i \rbrace$ *is the set of strategies available to him.*

*A strategy profile*$s=(s_1, . . . , s_n)$ *is an ordered set consisting of one strategy for each of the n players in the game.*

**Dominant strategy**: *A strategy* $s^{*}_{i}$ *is dominant (distinguished by the* $*$) *if for each player *$i \in N$ *and each strategy *$s_{i \in S_i}$, *the following inequality is satisfied: *

1 $$\begin{aligned} \forall i, \quad \Pi _{i}(s^{*}_{i}, s_{j}) \ge \Pi _{i}(s_{i}, s_{j}), \quad \forall s^{*}_{i}. \end{aligned}$$

*Analogously we can define a dominant strategy for other players* $s_{j}^{*}$.

**Payoff**: *By the payoff for the i-th player *$\Pi _{i}(s_1, . . . , s_n)$, *we mean either:*

1. *The utility player* *i receives after all players have picked their strategies and the game has been played out; or*
2. *The expected utility he receives as a function of the strategies chosen by himself and the other players*.

**Outcome**: *The outcome of the game is a set of interesting elements that the modeler picks from the values of actions, payoffs, and other variables after the game is played out.*

**Game Matrix**: *The Game Matrix or the normal form consists of*

1. *All possible strategy profiles* $s_1, s_2, . . . , s_n$.
2. *The outcome of payoff functions* $\Pi _{i}$* mapping onto strategies* $s_i,(i=1,2,...,n)$.

**Game**: *A game is an ordered triple* $G= (N, (S_i)_{i \in N}, (\Pi _i)_{i \in N})$, *in which:* $N = {1, 2, . . . , n}$ *is a finite set of players.*

$S_i$ *is the set of strategies of player i*, *for every player *$i \in N$.

*We denote the set of all strategy profiles by*$S = S_1 \times S_2 \times \cdots \times S_n$.

$\Pi _i : S \longrightarrow {\mathbb {R}}$ *is a function associating each strategy profile* $s = (s_i)_{i\in N}$ *with the payoff (= utility)* $\Pi _i(s)$ *to player i*, *for every player *$i \in N$.

**Equilibrium**: *An equilibrium *$s^{*} = (s^{*}_{1}, . . . , s^{*}_{n})$ *is a strategy profile consisting of the best strategy for each of the n players in the game.*

**Nash equilibrium (NE)**: *The strategy profile* $s^{*}=(s_1^{*},..., s_{n}^{*})$ *is a NE if for each player* $i \in N$ *and each strategy* $s_{i \in S_i}$ *the following is satisfied:*

2 $$\begin{aligned} \forall i, \quad \Pi _{i}(s^{*}_{i}, s^{*}_{j}) \ge \Pi _{i}(s_{i}, s^{*}_{j}), \quad \forall s^{*}_{i}. \end{aligned}$$

*The payoff* $\Pi _{i}(s^{*}_{i}, s^{*}_{j})$ *is the equilibrium payoff corresponding to the NE for the strategy* $s^{*}_{j}$.

**Pareto optimal (PO)**: *The Pareto Optimal is the outcome in which no player can obtain a higher payoff without reducing the payoff of another player.*

## Traditional approach in QG

### Classical approach

We have that the bimatrix for Prisoner’s Dilemma and Battle of the Sexes (see Eq. (8))3with definite values for the entries. These moves are chosen without any form of communication between the players. The set of strategies are $S_k=\lbrace C, D \rbrace$ for $k \in A, B$ that label the players, Alice and Bob, respectively.

The *Prisoner’s Dilemma* (PD) illustrates the situation where players, who have committed a crime together, are interrogated in separate cells. The two possible moves for each player are to cooperate (*C*), that is, not to confess the crime, or defect (*D*), confess the crime.

The entries of the table represent all possible moves where players can cooperate with payoff (3, 3), for which they both get the lowest possible sentence, or one player can cooperate and the other defect with a payoff are (5, 0) or (0, 5). Here the defector gets no sentence (highest possible payoff), while the cooperator gets the highest possible sentence (lowest payoff). The other possible case is when both players defect with payoff (1, 1). In this case they both get an intermediate sentence (a lower payoff than if they both cooperate). Although cooperating results in an overall better outcome for both players, a suboptimal solution can be chosen because of distrust between players. Now, regarding the payoffs defined in (3), we have that *D* is the more probable strategy for the players, therefore, thinking rationally and distrusting the other player, both players tend to choose to defect *D*. This is the result (*D*, *D*). This choice is globally suboptimal because the players could have had a better outcome by cooperating. This is the dilemma of the game.

The strategy (*C*, *C*) is the Pareto Optimal (PO). Any deviation from this strategy by one player, that improves his payoff, leads to a lowering of the payoff of the other. If player *A* tries to increase his payoff, the only option is (5, 0), so that the other player looses payoff. Starting from any other strategy, the player either cannot increase his payoff or both players can achieve a better payoff $((1,1)\rightarrow (3,3))$. Formally, the strategy (*C*, *C*) is PO because it is not a dominant strategy, that is, $\Pi _A(C,S_k) \ngeq \Pi _A(S_k,S_k)$ or $\Pi _B(S_k, C) \ngeq \Pi _B(S_k,S_k)$. Therefore, the definition (1) is not satisfied for this strategy.

The usual exposition of the *Battle of the Sexes* (BS) is that one player, Alice, is fond of the opera, while Bob prefers to watch TV, but they want to spend the night together. In the absence of communication, they have a dilemma, and choose their strategies. In the game matrix, *O* and *T* represent Opera and TV respectively. There are two NE (*O*, *O*) and (*T*, *T*) where no player can increase their payoff by changing only their strategy while the others keep theirs. The first (*O*, *O*) is advantageous for Alice and the second (*T*, *T*) is favorable to Bob.

### Eisert’s approach: the quantum game

Here we explain Eisert’s approach to QG for the Prisoner’s Dilemma (PD)^6,7^. The payoff matrix is given by4and for the PD we have

5 $$\begin{aligned} \eta _{1}^{A}=\eta _{1}^{B}=3 \quad ; \quad \eta _{2}^{A}=\eta _{3}^{B} =0 \quad ; \quad \eta _{3}^{A}=\eta _{2}^{B} =5 \quad ; \quad \eta _{4}^{A}=\eta _{4}^{B}=1. \end{aligned}$$

In contrast to the classical approach, Eisert characterises the game by the strategies corresponding to quantum operations, in which the classical strategies are included (to defect (D) and to cooperate (C)).

The first and second set of strategies involve quantum operations which are local rotations with one and two parameters. The matrix representation of the corresponding unitary operators is represented by

6 $$\begin{aligned} U(\theta )= \left( \begin{array}{cc} \cos \frac{\theta }{2} &{} \sin \frac{\theta }{2} \\ -\sin \frac{\theta }{2} &{} \cos \frac{\theta }{2} \\ \end{array} \right) , \qquad U(\theta , \phi )= \left( \begin{array}{cc} e^{i\phi }\cos \frac{\theta }{2} &{} \sin \frac{\theta }{2} \\ -\sin \frac{\theta }{2} &{} e^{-i\phi }\cos \frac{\theta }{2} \\ \end{array} \right) . \end{aligned}$$

In this case, the unitary operations are of one parameter. Selecting strategies amounts to choosing two angles $\theta _A$ and $\theta _B$^7^. In this game, the above operation coincides with the classical version of the Prisoner’s Dilemma; the pay-offs are identical to the payoffs in the classical Prisoner’s Dilemma with mixed strategies, where cooperation is chosen with the probabilities $P = \cos ^{2} (\theta _A / 2)$ and $Q = \cos ^{2} (\theta _A / 2)$^7^.

A strategy means the choice of appropriate angles $\theta _A$, $\phi _A$ and $\theta _B$, $\phi _B$^6,7^. The classical strategies can be performed when $C \sim U(0,0)$ and $D \sim U(\pi ,0)$. The Nash equilibrium (*D*, *D*) is no longer an equilibrium solution, as both players can benefit from deviating from the *D* strategy. However, with the disappearance of the classical solution, another NE arises, given by $({\mathcal {Q}}, {\mathcal {Q}})$. The ${\mathcal {Q}}$ strategy is associated with the operation

7 $$\begin{aligned} {\mathcal {Q}} \sim U_A(0, \pi /2)= U_B(0, \pi /2)= \left( \begin{array}{cc} i &{} 0\\ 0 &{} -i\\ \end{array} \right) . \end{aligned}$$

This NE is unique and serves as the only acceptable solution to the game. The surprising fact is that $\Pi _{A} ({\mathcal {Q}}, {\mathcal {Q}}) = \Pi _{B} ({\mathcal {Q}}, {\mathcal {Q}}) = 3$, instead of 1. Thus, the quantum strategy ${\mathcal {Q}} \sim U(0, \pi /2)$ emerges as the new equilibrium when both players have access to the two-parameter set of Eq. (6) of unitary operators^6,7^. In a classical game, no player can win without lowering another player’s expected pay-off. In this sense, one can say that the dilemma of the original game has completely disappeared, i.e, the dilemma of the game is broken.

In this sense, quantum entanglement allows breaking the dilemma of the game, since a maximally entangled state has been used, contrary to the classical case where the dilemma persists. Therefore, the incorporation of an entangled state in the initial state of the game for the set of two-parameter strategies (quantum game), generates strategies that are not available to the players in the set of one-parameter strategies (classical game).

In later work by Benjamin and Hayden^8^, they comment on Eisert’s work on the Prisoner’s Dilemma and find that when the two-parameter set is extended to include all local unitary operations, the strategy ${\mathcal {Q}}$ is not an *original equilibrium*, because ${\mathcal {Q}}$ is not in the original set of game strategies^6,8^. Also, in reference^8^ they observed that the set of two-parameter quantum strategies is not closed under composition, although this closure is a necessary requirement for any set of quantum game strategies. This means that Eisert’s approach^6^, allowed both players the same strategy set but introduced an arbitrary constraint into that set. This amounts to allowing for a certain strategy while forbidding the logical counter-strategy which one would expect in a game. So Ref.^8^ shows that ${\mathcal {Q}}$ emerges as the ideal strategy only because the strategy set is restricted arbitrarily. The type of quantum strategies, such as ${\mathcal {Q}}$, is known in QG as non-local strategies.

For Eisert’s approach to QG for the Battle of the Sexes, the dilemma is not broken, because the quantum strategy ${\mathcal {Q}} \sim U(0, \pi /2)$ has the same NE than in the classical version. However, for another version of QG, the dilemma game is broken, (see Ref.^14^.)

## Non-factorizable probabilities for quantum games

The approach of joint probabilities for QG, posed by Iqbal et al^22^, considers four probabilities in the player pay-off relations. It is a valid question to ask if the pay-off relations in the QG can be described as mixed-strategy pay-off relations in the classical game, that is, can we write pay-off relations for the quantum game in terms of joint probabilities?. To this end, in Ref.^22^, they use pay-off relations that describe the factorizability of joint probabilities through a set of equations that connect player strategies with a probability distribution of player pay-offs. In this way, a quantum game can then be described as the game in which non-factorizable probabilities are the norm^22^. A bimatrix description of the game is considered8Where Alice and Bob are the players, with their strategies $S_{1}, S_{2}$ and $S_{1}^{'},S_{2}^{'}$ respectively and $\eta _{1}^{k}, \eta _{2}^{k}, \eta _{3}^{k}, \eta _{4}^{k}$ where $k \in A, B$, corresponding to payoffs for each strategies of players. For this bimatrix (8), if Alice and Bob take one strategy, e.g. $(S_{2}, S_{1}^{'})$, their payoffs are $(\eta _{3}^{A}, \eta _{3}^{B })$, similarly, it happens for the other strategies. With the latter in mind, the pay-off relations for classical games are

9 $$\begin{aligned} \Pi _{k}(P,Q)= \eta _{1}^{k} PQ +\eta _{2}^{k} P(1-Q) +\eta _{3}^{k} (1-P)Q +\eta _{4}^{k} (1-P)(1-Q), \end{aligned}$$

where $P, Q \in [0, 1]$. The values of these probabilities indicate the choice of a particular strategy for the players. For example, If Alice and Bob choose the strategy $(S_{2}, S_{1}^{'})$ their payoffs are $(\eta _{3}^{A}, \eta _{3}^{B })$ according to (8). Therefore, the value of the probabilities are $P=0$ and $Q=1$. We can write the previous relation for a QG as

10 $$\begin{aligned} \Pi _{k}(U_{A},U_{B})= \eta _{1}^{k} \epsilon _{1} +\eta _{2}^{k} \epsilon _{2} +\eta _{3}^{k} \epsilon _{3} + \eta _{4}^{k} \epsilon _{4}, \end{aligned}$$

where

11 $$\begin{aligned} \epsilon _{1} = \vert \langle S_{1}S_{1}^{'} \vert \psi _{f} \rangle \vert ^{2} \quad ; \quad \epsilon _{2} = \vert \langle S_{1}S_{2}^{'} \vert \psi _{f} \rangle \vert ^{2} \quad ; \quad \epsilon _{3} = \vert \langle S_{2}S_{1}^{'} \vert \psi _{f} \rangle \vert ^{2} \quad ; \quad \epsilon _{4} = \vert \langle S_{2}S_{2}^{'} \vert \psi _{f} \rangle \vert ^{2}, \end{aligned}$$

are four probabilities obtained by projecting the final quantum state $\epsilon _{f}$ of the game onto the four basis vectors $\vert S_{1}S_{1}^{'} \rangle$, $\vert S_{1}S_{2}^{'} \rangle$, $\vert S_{2}S_{1}^{'} \rangle$, $\vert S_{2}S_{2}^{'} \rangle$^6,7^. The probabilities $\epsilon _{i}$ are appropriately normalized as $\sum _{i=1}^{4} \epsilon _{i}=1.$

Comparing the pay-off relations in the classical game (9) and in the quantum game (10), we note that the pay-off relations in the quantum game can be reduced to the pay-off relations in the classical game when probabilities $\epsilon _{i}$ are factorizable. Probabilities $\epsilon _{i}$ are factorizable when for a given set of values assigned in range [0, 1] to probabilities $\epsilon _{i}$, we can find two probabilities $P,Q \in [0, 1]$ such that $\epsilon _{i}$ can be written in terms of *P* and *Q* as

12 $$\begin{aligned} \epsilon _{1} = PQ \quad ; \quad \epsilon _{2} =P(1-Q) \quad ; \quad \epsilon _{3} =(1-P)Q \quad ; \quad \epsilon _{4} = (1-P)(1-Q). \end{aligned}$$

In the previous case, for the probabilities $\epsilon _{i}$, we can associate the probabilities *P* and *Q* to the players, so that the pay-off relations in the quantum game (see Eq. (10)) are interpreted as corresponding to a mixed-strategy classical game, i.e.

13 $$\begin{aligned} \Pi _{k}P,Q)=\Pi _{k}(U_{A},U_{B}), \end{aligned}$$

with *P* and *Q* satisfying the factorizability relations (12). In (12), $\epsilon _{i}(P,Q)$ represents not just a specific set of four numbers in [0, 1] that add up to 1, but the entirety of such four numbers that can be generated by the quantum mechanical set-up used for playing a quantum game.

The probabilities $\epsilon _{i}$, however, may not be factorizable in the sense described by Eq. (12). That is, the measure of a quantum game can result in such probabilities $\epsilon _{i}$ $(0 \le \epsilon _{i} \le 1)$ that one cannot find $P,Q \in [0, 1]$ so that Eqs. (12) are satisfied. Viewing the pay-off relations (10) from this probabilistic viewpoint, it then seems natural to ask whether we can obtain the pay-offs (10) by simply removing the factorizability relations (12), and if the case, then what are the possible new outcomes of the game.

At this point, we note that when the $\epsilon _{i}$ are factorizable, in the sense described by Eq. (12), that gives the relationship between *P*, *Q* and $\epsilon _{i}$. Using these relations, the player strategies *P* and *Q* and the terms of the probabilities $\epsilon _ {i}(P, Q)$ can be related as

14 $$\begin{aligned} P(\epsilon _{i}) = \epsilon _{1}(P,Q) +\epsilon _{2}(P,Q) \quad ; \quad Q(\epsilon _{i}) = \epsilon _{1}(P,Q) +\epsilon _{3}(P,Q), \end{aligned}$$

then using the expressions in (11), we can write the previous relations as

15 $$\begin{aligned} P(\epsilon _{i}) =\vert \langle S_{1}S_{1}^{'} \vert \psi _{f} \rangle \vert ^{2} +\vert \langle S_{1}S_{2}^{'} \vert \psi _{f} \rangle \vert ^{2} \quad ; \quad Q(\epsilon _{i})=\vert \langle S_{1}S_{1}^{'} \vert \psi _{f} \rangle \vert ^{2} +\vert \langle S_{2}S_{1}^{'} \vert \psi _{f} \rangle \vert ^{2}, \end{aligned}$$

where $P(\epsilon _{i})$ and $Q(\epsilon _{i})$ are what we call the game probabilities. Each player has the freedom to play the strategies *P* and *Q*, and these are considered independent of each other.

This motivates to consider $\epsilon _{1}$ as a function of the game probabilities $P(\epsilon _{i})$ and $Q(\epsilon _{i})$ more general than above, i.e. $\epsilon _{1} = \epsilon _{1}(P, Q)$. It is this function of the game probabilities that maps the pair (*P*, *Q*) to the interval [0, 1]. Note that for the quantum game, the players’ strategies are a unitary transformations $U_{A}$ and $U_{B}$, whereas for the new game defined by pay-off relations, the players’ strategies are $P, Q \in [0, 1]$. The game defined by the pay-off relations makes no reference to the quantum operations and can thus simply be called a non-factorizable game or a game that allows non-factorizable probabilities.

Thus, from comparing and rewriting the expressions corresponding to the payoffs for classical and QG, we obtain the functions $\epsilon _{2}(P, Q)$, $\epsilon _{3}(P, Q)$ and $\epsilon _{4}(P, Q)$ defined as

16 $$\begin{aligned} \epsilon _{2}(P, Q) = P(\epsilon _{i}) -\epsilon _{1}(P, Q) \quad ; \quad \epsilon _{3}(P, Q) = Q(\epsilon _{i}) -\epsilon _{1}(P, Q) \quad ; \quad \epsilon _{4}(P, Q) = 1 -[(P(\epsilon _{i})+Q(\epsilon _{i}))-\epsilon _{1}(P, Q)]. \end{aligned}$$

The functions $\epsilon _{2}(P, Q)$, $\epsilon _{3}(P, Q)$ and $\epsilon _{4}(P, Q)$ produce values within the range [0, 1]. Of course, being $\epsilon _{1}(P, Q)=PQ$, one will have a classical game. In view of the above, for a game where this constraint is not satisfied, it can be called a non-factorizable game or a game that allows non-factorizable probabilities. These type of games are called a quantum game since they entail non-factorizable probabilities^20,21^. For a game with non-factorizable probabilities, it is required that $\epsilon _{1}(P,Q)$, satisfy

17 $$\begin{aligned} \epsilon _{1}(P, Q)\le PQ. \end{aligned}$$

The expressions for $P(\epsilon _{i})= \epsilon _{1}(P,Q) +\epsilon _{2}(P,Q)$ and $Q(\epsilon _{i})= \epsilon _{1}(P,Q) +\epsilon _{3}(P,Q)$ connect the player strategies with the probabilities $\epsilon _{i}$. Note that the expressions obtained for $P(\epsilon _{i})$ and $Q(\epsilon _{i})$ are simplest for the classical strategies^22^. However, for the game with non-factorizable probabilities, these expressions will not be simple, since for $P(\epsilon _{i})$ and $Q(\epsilon _{i})$, the probabilities in $\epsilon _{i}$ are correlated, as will be discussed in the next section. On the other hand, it is important to note that for this game^22^, the strategies of the players are $P, Q \in [0, 1]$ and not the unitary transformations $U_{A}$ and $U_{B}$^6^, i.e. these strategies do not correspond to quantum operations.

## New approach to QG

### Iqbal’s approach for joint probabilities in QG

In the previous section we formulated QG in terms of joint probabilities. We can write the previously defined parameters $\epsilon _{i}$ to determine the NE for Eisert’s QG^6,7^. The pay-offs in the latter games are associated with two versions of game, according to the set of strategies that players use as: (i) *quantum game with classical strategies* or one-parameter set of strategies and (ii) *quantum game with quantum strategies* or two-parameter set of strategies^7^, where the quantum strategies are represented by the super-index $\star$.

Based on the description of the joint probabilities for QG, we identify^6,7^ a quantum game with classical strategies where the parameters $\epsilon _{i}$ are

18 $$\begin{aligned} \epsilon _1=c_A^2c_B^2 \quad ; \quad \epsilon _2=c_A^2(1-c_B^2) \quad ; \quad \epsilon _3=(1-c_A^2)c_B^2 \quad ; \quad \epsilon _4= (1-c_A^2)(1-c_B^2), \end{aligned}$$

and the game probabilities

19 $$\begin{aligned} P=\epsilon _1+\epsilon _2 \quad ; \quad Q=\epsilon _1+\epsilon _3, \end{aligned}$$

where

20 $$\begin{aligned} p \equiv c_A^2=\cos ^2\left( \frac{\theta _A}{2}\right) \quad ; \quad q \equiv c_B^2=\cos ^2\left( \frac{\theta _B}{2}\right) , \end{aligned}$$

and $p, q \in [0, 1]$. Using these definitions in (18), we have

21 $$\begin{aligned} \epsilon _1=pq \quad ; \quad \epsilon _2=p(1-q) \quad ; \quad \epsilon _3=(1-p)q \quad ; \quad \epsilon _4=(1-p)(1-q), \end{aligned}$$

so, we explicitly arrive at the game probabilities

22 $$\begin{aligned} P=p \quad ; \quad Q=q. \end{aligned}$$

From the expressions obtained for $\epsilon _{1}$, we observe that QG with classical strategies are classical games, recovering in this way, the same results of Ref.^22^. On the other hand, if we identify the parameters $\epsilon _{i}^{\star }$ in a QG with quantum strategies, from^6,7^

23 $$\begin{aligned} \epsilon _1^{\star }=\vert \cos (\phi _A +\phi _B)c_A c_B \vert ^2 \quad ; \quad \epsilon _2^{\star }= \vert \cos (\phi _A)c_A s_B -\sin (\phi _B)s_A c_B\vert ^2 \end{aligned}$$

24 $$\begin{aligned} \epsilon _3^{\star }= \vert \sin (\phi _A)c_A s_B -\cos (\phi _B)s_A c_B \vert ^2 \quad ; \quad \epsilon _4^{\star }= \vert \sin (\phi _A +\phi _B)c_A c_B +s_A s_B \vert ^2 \end{aligned}$$

where $s_A \equiv \sin (\theta _{A}/2 )$ and $s_B \equiv \sin (\theta _{B}/2 )$. The probabilities for a QG are then written as

25 $$\begin{aligned} P^{\star }=\epsilon _1^{\star }+\epsilon _2^{\star } \quad ; \quad Q^{\star }=\epsilon _1^{\star }+\epsilon _3^{\star }. \end{aligned}$$

We can identify from the expressions in (23) and (24), that the joint probabilities are not factorizable, since we have correlations between the angles $\phi _{k}$ and $\theta _{k}$ for $k=A,B$.

So far, we can determine that the game probabilities $P^{\star }$ and $Q^{\star }$ are correlated for the strategies of the players, as manifested through the corresponding angles. We see that the parameters $\epsilon _i^{\star }$ are the ones that produce the correlations in the probabilities $P^{\star }$ and $Q^{\star }$^22^. As expected $P^{\star }$ and $Q^{\star }$ are non local^8,22^, because the probabilities for a quantum game are dependent on the parameters of the unitary transformations ($U_ {A}, U_ {B}$).

### Joint probabilities in QG, eliminating non-local strategies

Now we propose to describe QG from their joint probabilities, explicitly freeing the description from non-local strategies.To this end, we describe QG through the probabilities $\epsilon _i^{\star }$, $P^{\star }$ and $Q^{\star }$ as functions only of the joint probabilities. This is a novel result, since we generalize the description discussed by^22^, eliminating non-local strategies. For this, it is necessary to define the following probabilities

26 $$\begin{aligned} p^{\star } \equiv \cos ^2(\phi _A) \quad ; \quad q^{\star } \equiv \cos ^2(\phi _{B}). \end{aligned}$$

where $p^{\star }, q^{\star } \in [0, 1]$, these probabilities are inspired by Eq. (20). Next, the probabilities defined in (20) and (26) are introduced into the expressions (23) and (24),

27 $$\begin{aligned} \epsilon _{1}^{\star }= & {} [2p^{\star }q^{\star }-(p^{\star }+q^{\star })+1]pq -\gamma _{\epsilon _{1}^{\star }} ,\end{aligned}$$

28 $$\begin{aligned} \epsilon _{2}^{\star }= & {} p^{\star }p-q^{\star }q+q-(1+p^{\star }-q^{\star })pq-\gamma _{\epsilon _{2}^{\star }},\end{aligned}$$

29 $$\begin{aligned} \epsilon _{3}^{\star }= & {} -(p^{\star }p-q^{\star }q)+p-(1-p^{\star }+q^{\star })pq-\gamma _{\epsilon _{3}^{\star }},\end{aligned}$$

30 $$\begin{aligned} \epsilon _{4}^{\star }= & {} (p^{\star }+q^{\star }-2p^{\star }q^{\star }+1)pq+1-(p+q)+\gamma _{\epsilon _{1}^{\star }}+\gamma _{\epsilon _{4}^{\star }} \end{aligned}$$

where $$\gamma _{\epsilon _{1}^{\star }}=2pq\sqrt{p^{\star }q^{\star }(1-p^{\star })(1-q^{\star })} \quad , \quad \gamma _{\epsilon _{2}^{\star }}=\gamma _{\epsilon _{3}^{\star }}=2\sqrt{pq(1-p)(1-q)p^{\star }(1-q^{\star })}$$ and

$$\gamma _{\epsilon _{4}^{\star }}=2\sqrt{pq(1-p)(1-q)}\left[ \sqrt{(1-p^{\star })q^{\star }} +\sqrt{p^{\star }(1-q^{\star })} \right] $$.

Then the quantum game probabilities are (see 25)

31 $$\begin{aligned} P^{\star }=p^{\star }p-q^{\star }q+q-2p^{\star }(1-q^{\star })pq-\gamma _{\epsilon _{1}^{\star }}-\gamma _{\epsilon _{2}^{\star }}, \end{aligned}$$

and

32 $$\begin{aligned} Q^{\star }=-(p^{\star }p-q^{\star }q)+p-2(1-p^{\star })q^{\star }pq-\gamma _{\epsilon _{1}^{\star }}-\gamma _{\epsilon _{3}^{\star }}. \end{aligned}$$

To obtain the equilibrium of the game, we impose the conditions for the Nash equilibrium and Pareto optimal, so that

33 $$\begin{aligned} p=q \equiv w \quad ; \quad p^{\star }=q^{\star } \equiv u . \end{aligned}$$

This way, we can use these definitions in expressions $\gamma _{\epsilon _{1}^{\star }}$, $\gamma _{\epsilon _{2}^{\star }}$, $\gamma _{\epsilon _{3}^{\star }}$ and $\gamma _{\epsilon _{4}^{\star }}$, so the parameters $\epsilon _{1}^{\star }$, $\epsilon _{2}^{\star }$, $\epsilon _{3}^{\star }$ and $\epsilon _{4}^{\star }$, can be written as

34 $$\begin{aligned} \epsilon _{1}^{\star }=(1-2u)^{2}w^{2} \quad ; \quad \epsilon _{2}^{\star }=\epsilon _{3}^{\star }=w(1-w)\left[ 1-2\sqrt{u(1-u)}\right] , \end{aligned}$$

and

35 $$\begin{aligned} \epsilon _{4}^{\star }= \left[ 1+4u(1-u) \right] w^{2} +1-2w +4w(1-w)\sqrt{u(1-u)}. \end{aligned}$$

The game probabilities are then

36 $$\begin{aligned} P^{\star }=w-4u(1-u)w^{2}-2w(1-w)\sqrt{u(1-u)}, \end{aligned}$$

37 $$\begin{aligned} Q^{\star }=w-4u(1-u)w^{2}-2w(1-w)\sqrt{u(1-u)}. \end{aligned}$$

In the expressions (34) to (37), we arrived at a new description of Eisert QG from their joint probabilities; a quantum game with quantum strategies. From this description, we can determine directly the equilibria of the QG. This procedure is analogous to the infinitely repeated quantum games^27^, where there are conditions on the probabilities (strategies) to obtain the equilibria of the game. We have thus generalized the description discussed by^22^.

Non-local strategies have no place in our approach because the set of all strategies are available in the rules of the game. The set is defined by the probabilities *p* and *q* (set for classical games) and the probabilities $p^{\star }$ and $q^{\star }$, therefore there is no arbitrariness in the strategies. This makes a quantum game with quantum strategies a description that is more robust and general.

Returning, to the non-factorizability of the joint probabilities; we have the term $\epsilon _{1}^{\star }$ in expression (34) where it is explicit that QG are characterized by joint non-factorizable probabilities, meaning that the correlations are due to entanglement. Furthermore, the approach we took to determine the factorizability of the $\epsilon _{1}^{\star }$ parameter was the one associated with the probabilities *p* and *q* and $p^{\star }$ and $q^{\star }$, due to the existing correlations. Particularly, non-factorizability was considered in the probabilities represented by $w \equiv p=q$ and $u \equiv p^{\star }=q^{\star }$, since $\epsilon _{1}^{\star }$ is not just a product of *w* and *u* as for classical games.

### Nash equilibrium in joint probabilities for QG

In view of the results obtained in the previous section and in order to obtain a complete description of QG through their joint probabilities, it is necessary to determine the NE inequalities corresponding to our approach. For this purpose, we use the inequalities,

38 $$\begin{aligned} \Pi _A(P^{NE},Q^{NE}) -\Pi _A(P,Q^{NE}) \ge 0 \quad ; \quad \Pi _B(P^{NE},Q^{NE}) -\Pi _B(P^{NE},Q) \ge 0, \end{aligned}$$

where the strategy $(P^{NE},Q^{NE})$ defines NE.

With these expressions in mind, we write the pay-offs for Alice and Bob, when players use classical strategies. Starting from the parameters $\epsilon _{i}$ of Eq. (18), pay-offs can be written as

39 $$\begin{aligned} \Pi _k(p,q)= \eta _1^k pq +\eta _2^k p(1-q) +\eta _3^k (1-p)q +\eta _4^k (1-p)(1-q), \end{aligned}$$

for $k \in A,B$. Thus, from (38) and using the pay-offs (39), we get the following expressions

40 $$\begin{aligned}{}[(\eta _1^A -\eta _2^A -\eta _3^A +\eta _4^A)q^{NE} +\eta _2^A -\eta _4^A](p^{NE}-p) \ge 0, \end{aligned}$$

41 $$\begin{aligned}{}[(\eta _1^B -\eta _2^B -\eta _3^B +\eta _4^B) p^{NE} +\eta _3^B -\eta _4^B](q^{NE}-q)\ge 0. \end{aligned} $$

These inequalities are what the NE must satisfy, which corresponds to the strategy $(p^{NE},q^{NE})$.

Analogously to the classical case, we use Eqs. (34) and (35) for the quantum pay-offs for players

42 $$\begin{aligned}&\Pi _k (w,u)= \eta _1^k( 1-2u)^2 w^2 +(\eta _2^k +\eta _3^k)w(1-w)\left[ 1-2\sqrt{u(1-u)} \right] \nonumber \\&\quad +\eta _4^k\left\{ \left[ 1 +4u(1-u) \right] w^2 +1-2w +4w(1-w)\sqrt{u(1-u)} \right\} . \end{aligned}$$

Using the conditions for the NE (38) and the pay-offs (42), we obtain

43 $$\begin{aligned}&\left\{ \left[ (\eta _{1}^{A} +\eta _{4}^{A}) -4(\eta _{1}^{A} -\eta _{4}^{A})u^{NE}(1-u^{NE})\right] (w^{NE} +w) -2\eta _4^A \right. \nonumber \\&\qquad {} + \left. \left[ \eta _{2}^{A} +\eta _{3}^{A} -2(\eta _{2}^{A} +\eta _{3}^{A} -2\eta _{4}^{A})\sqrt{u^{NE}(1-u^{NE})}\right] (1-w^{NE} -w) \right\} (w^{NE} -w) \ge 0, \end{aligned}$$

and

44 $$\begin{aligned} 4&(\eta _{1}^{A} -\eta _{4}^{A})(u^{NE} -u)(u^{NE} +u -1)(w^{NE})^{2} \nonumber \\&\qquad {} +2(\eta _{2}^{B} +\eta _{3}^{B} -2\eta _{4}^{B})w^{N}(1- w^{NE})\left[ \sqrt{u(1-u)} -\sqrt{u^{NE}(1-u^{NE})} \right] \ge 0. \end{aligned}$$

The above inequalities must be satisfied in the strategy $(w^{NE},u^{NE})$ which corresponds to the NE.

## Information capacity of a quantum game from the joint probabilities

Information in QG has been developed in different works^23–25^, discussed previously. In these contributions the density operator is the main protagonist in order to determine the game information. However, as we have discussed above, we focus on the joint probabilities to determine the quantum game information.

Likewise, in the previous sections, we mentioned that the games are characterized by the nature of their joint probabilities: if these are factorizable, you have a classical game, otherwise, we have a quantum game, the latter reflecting entanglement. Thus, through the definition of information that we propose below, we can quantify the entanglement in QG. It is necessary to measure the information in the game with Shannon’s entropy since we studied the information through joint probabilities. For the purpose of quantifying the information capacity, we begin our assessment from the results obtained in Sects. 4, 5 and the capacity definitions for classical and quantum channels^1^.

We define the capacity of a game as follows: The *player information* is defined as ${\mathcal {I}}_{G} \equiv \ \frac{1}{2} [H(P^{\alpha }_{x}) +H(Q^{\alpha }_{x})]$, where $\alpha$ is the super-index corresponding to the game under consideration, the sub-index being $x=A, B$ corresponding to the players Alice and Bob. On the other hand, *H*(.) corresponds to the binary entropy, which is defined as $H(P)\equiv -P \, \log P -(1-P) \, \log (1-P)$, where logarithms are taken base two. The players information is maximum or minimum when $H(P^{\alpha }_{x})$ and $H(Q^{\alpha }_{x})$ are maximum or minimum respectively. In this way, we have that the *game information* for each player as

45 $$\begin{aligned} {\mathcal {G}}^{\alpha }_{x} \equiv {\mathcal {I}}_{G} +H(\epsilon _{1}^{\alpha }) -H(\epsilon _{4}^{\alpha })= \frac{1}{2} [H(P^{\alpha }_{x}) +H(Q^{\alpha }_{x})] +H(\epsilon _{1}^{\alpha }) -H(\epsilon _{4}^{\alpha }). \end{aligned}$$

In the above expression, we understand the game as an information channel, therefore we have that the game information is the sum of the input and output information of the game represented by ${\mathcal {I}}_{G}=\frac{1}{2} [H(P^{\alpha }_{x}) +H(Q^{\alpha }_{x})]$ and $H(\epsilon _{1}^{\alpha })$ respectively, minus the information of the correlation between them given by $H(\epsilon _{4}^{\alpha })$. In this way, we define the input probabilities of a game as $P^{\alpha }_{x}$ and $Q^{\alpha }_{x}$, and the output probabilities as $\epsilon _{1}^{\alpha }$. The existing correlations between the input and output probabilities of the game, are given by $\epsilon _{4}^{\alpha }$, since $\epsilon _{4}^{\alpha }$ relates to $P^{\alpha }_{x}$, $Q^{\alpha }_{x}$ and $\epsilon _{1}^{\alpha }$, according to the expression $\epsilon _{4}^{\alpha } = 1 -[(P^{\alpha }_{x}+Q^{\alpha }_{x})-\epsilon _{4}^{\alpha }]$(see 16).

For a game where the probabilities $P^{\alpha }_{x}$ and $Q^{\alpha }_{x}$ are not correlated (a classical game), the information defined by (45) coincides with the properties of the mutual information (the information capacity in Shannon’s theory), only for the game information of each player. From the definition of information in (45), we can rewrite the difference between the terms $H(\epsilon _{1}^{\alpha })$ and $H(\epsilon _{4}^{\alpha })$, as ${\mathcal {I}}_{G}\mid {\mathcal {I}}_{\Pi ^{\alpha }} \equiv H(\epsilon _{4}^{\alpha })-H(\epsilon _{1}^{\alpha })$, where this represents the information we know about the game when we have the output information. This makes ${\mathcal {I}}_{G}\mid {\mathcal {I}}_{\Pi ^{\alpha }}$ equivalent, in meaning, with the conditional entropy in Shannon’s theory, but not in the algebraic sense, due to correlations in the joint probabilities. Thus, we can rewrite the expression (45) as

46 $$\begin{aligned} {\mathcal {G}}^{\alpha }_{x} \equiv {\mathcal {I}}_{G} -{\mathcal {I}}_{G}\mid {\mathcal {I}}_{\Pi ^{\alpha }}= \frac{1}{2} [H(P^{\alpha }_{x}) +H(Q^{\alpha }_{x})] +H(\epsilon _{1}^{\alpha }) -H(\epsilon _{4}^{\alpha }). \end{aligned}$$

The game information is an average of the information of the players, therefore the *game information* can be written as

47 $$\begin{aligned} {\mathcal {G}}^{\alpha } \equiv \frac{1}{2} {\mathcal {G}}^{\alpha }_{x}. \end{aligned}$$

For games where ${\mathcal {I}}_{G}$ is the same for two-players, we will have ${\mathcal {G}}^{\alpha }={\mathcal {G}}^{\alpha }_{x}$. With the above definitions, we define the *Information Capacity of a Quantum Game from the Joint Probabilities* for an Eisert quantum game, (see Sect. 5) as

48 $$\begin{aligned} \chi ^{\alpha } \equiv \max _{P^{\alpha }_{x}, Q^{\alpha }_{x}} {\mathcal {G}}^{\alpha }, \end{aligned}$$

where ${\mathcal {G}}^{\alpha }$ is the game information, $P^{\alpha }_{x}$ and $Q^{\alpha }_{x}$ are the players probabilities in the game.

For the definition of the information capacity above, the input information is given by two probabilities, $P^{\alpha }_{x}$ and $Q^{\alpha }_{x}$, as in (45). This is not the case for Shannon information theory^1^, since the probabilities of entry are determined by a single probability, therefore there are no correlations.

The joint probabilities show the entanglement, through the correlations of the probabilities involved in (45) when the players use quantum strategies. At the same time, we will see below how this affects the information capacity in the games under consideration. When we determine the game information, we can quantify the entanglement through entropy.

## Joint probabilities approach and information in QG

In this section, we make use of the proposed formalism for joint probabilities and information in QG, particularly for Prisoner’s Dilemma and Battle of the Sexes. As we described before, to determine the best strategies that allow a solution (break the dilemma) to the game we must determine the NE. We now proceed to determine the NE through the expressions (40), (41), (43) and (44), when the players use classical and quantum strategies, considering the pay-offs for the game. In addition, we use the expression of information capacity proposed in Eq. (48) to determine information in the game. Finally, we determine the value of the pay-offs for Alice and Bob when the joint probabilities maximize and minimize the game information ${\mathcal {G}}^{\alpha }$.

### Nash equilibrium

The NE for the Prisoner’s Dilemma in the case of classical strategies is achieved for the strategy with a probability value $(p_{1}^{NE},q_{1}^{NE})=(0,0)$, since the expressions (40) and (41) hold for any value of *p* and *q*.

The NE for the PD, when contemplating quantum strategies^6,7^, is achieved for the probability values $(w^{NE}_{1},u^{NE}_{1})=(1,0)$ or $(w^{NE}_{2},u^{NE}_{2})=(1,1)$, since for both values, the inequalities in Eq. (43) and Eq. (44) are satisfied for any value of *w* and *u*, and the dilemma of the game disappears. The players have no motivation to deviate from these strategies. In the same way, when evaluating $(p_{1}^{NE},q_{1}^{NE}) \equiv (w^{NE*}_{0},u^{NE*}_{0})=(0,0)$, the NE for the game with classical strategies in the expressions in Eq. (43) and Eq. (44) are no longer the NE for the game.

For the Battle of the Sexes using classical strategies, the NE exists for the probability values $(p_{1}^{NE},q_{1}^{NE})=(0,0)$ and $(p_{2}^{NE},q_{2}^{NE})=(1,1)$. Because, the probabilities $(p_{1}^{NE},q_{1}^{NE})=(0,0)$ and $(p_{2}^{NE},q_{2}^{NE})=(1,1)$ in the inequalities Eqs. (40) and (41) hold for any value of *p* and *q*. However, when considering quantum strategies, we have an infinite number of Nash Equilibria^35^. Among these, there are ones that correspond to the following angles $\theta _{A}=\theta _{B}=\pi$ and $\phi _{A}=\phi _{B}=\pi /4$. This is equivalent to the probability values $(w^{NE}_{3},u^{NE}_{3})=(0,1/2)$, which satisfy the inequalities of Eqs. (43) and (44).

As a summary, we obtain the following equilibria for the classical and quantum strategies

**Table 1:** Nash equilibria in terms of joint probabilities.

Games	Classic Strategies	Quantum Strategies
Prisoner’s Dilemma	$(p_{1}^{{NE}},q_{1}^{{NE}})$	$(w^{{NE}}_{1},u^{{NE}}_{1}) \quad \vee \quad (w^{{NE}}_{2},u^{{NE}}_{2})$
Battle of the Sexes	$(p_{1}^{{NE}},q_{1}^{{NE}}) \quad \wedge \quad (p_{2}^{{NE}},q_{2}^{{NE}})$	$(w^{{NE}}_{3},u^{{NE}}_{3})$

The probability values for the NE are $(p_{1}^{NE},q_{1}^{NE})=(0,0)$, $(p_{2}^{NE},q_{2}^{NE})=(1,1)$ y $(w^{NE}_{1},u^{NE}_{1})=(1,0)$, $(w^{NE}_{2},u^{NE}_{2})=(1,1)$, $(w^{NE}_{3},u^{NE}_{3})=(0,1/2)$, for classical and quantum strategies respectively.

In the previous equations, we display the conditions for NE. The first interesting result, for our joint probabilities approach versus Eisert’s formulation^6,7^, is that we obtain an additional NE for the Prisoner’s Dilemma with quantum strategies, (which have the same pay-offs) and the dilemma is overcome. We can see in the set defined by the probabilities $u,w \in [0, 1]$, that the non-local strategies have no place in our approach as happens with Eisert’s approach, because the set of all strategies are available in the rules of the game. Therefore, there is no arbitrariness in the strategies. All strategies are contained in the set of strategies of the game. This is the main results of our work.

Interestingly, in the BS for $(w^{NE}_{3},u^{NE}_{3})$, the dilemma does not disappear, in contrast to what happens with the PD. The above is due to the symmetry of this particular game, since the game probabilities are $P^{\star }=Q^{\star }=\epsilon ^{\star }_{1}$ (because $\epsilon ^{\star }_{2}=\epsilon ^{\star }_{3}=0$, see Eq. (3)) contrary to that of the PD, where $P^{\star }=\epsilon ^{\star }_{1} +\epsilon ^{\star }_{2}$ and $Q^{\star }=\epsilon ^{\star }_{1} +\epsilon ^{\star }_{3}$. In the BS, even though entanglement generates a NE for quantum strategies, the game dilemma persists.

Notwithstanding the previous result, there is a way to use entanglement further to overcome the dilemma. To understand this answer we see what happens with the strategies of the games when the information is incorporated into the quantum games joint probabilities in the BS.

### Information in QG

With the definition of information capacity, proposed in the Eq. (48), the expression for $\chi ^{\alpha }$ corresponds to maximizing the game information on the probabilities $P^{\alpha }_{x}$ and $Q^{\alpha }_{x}$. These probabilities are obtained by maximizing the player information ${\mathcal {I}}_{G}$, since the information capacity consists of taking the channel information over the probabilities that maximize the input information. In this way, we can determine the information capacity for a quantum game $\chi ^{\alpha }$. For the PD, ${\mathcal {I}}_{G}$ is ${\mathcal {G}}^{\alpha }={\mathcal {G}}^{\alpha }_{A}={\mathcal {G}}^{\alpha }_{B}$ for the two-players since Eq. (45) can be used directly in Eq. (48). Then, the game information is maximized through the probabilities $P^{\alpha }_{x}$ and $Q^{\alpha }_{x}$, in order to obtain the information capacity. For the latter, we obtain the solutions of $P^{\alpha }_{x}$ and $Q^{\alpha }_{x}$, that maximize $H(P^{\alpha }_{x})$ and $H(Q^{\alpha }_{x})$ for each game, when the players employ the classical and quantum strategies.

Based on the definition of quantum information capacity for the PD, the relevant expressions for the probabilities $P^{\alpha }_{x}$ and $Q^{\alpha }_{x}$, and the parameters $\epsilon _{1}^{\alpha }$ and $\epsilon _{4}^{\alpha }$ we have

49 $$\begin{aligned} \chi ^{(PD)}&=\max _{P^{(PD)},Q^{(PD)}} {\mathcal {I}}_{G} -{\mathcal {I}}_{G}\mid {\mathcal {I}}_{\Pi ^{(PD)}}=2, \end{aligned}$$

50 $$\begin{aligned} \chi ^{(\star PD)}&=\max _{P^{\star (PD)},Q^{\star (PD)}} {\mathcal {I}}_{G} -{\mathcal {I}}_{G}\mid {\mathcal {I}}_{\Pi ^{(PD)}}=1, \end{aligned}$$

corresponding to the classical and quantum strategies, $\chi ^{(PD)}$ and $\chi ^{(\star PD)}$, respectively.

For the BS, the expression of the information capacity for the classical strategies is given by

51 $$\begin{aligned} \chi ^{(BS)}= \frac{1}{2}[\chi ^{(BS)}_{(A)}+\chi ^{(BS)}_{(B)}]=2-\frac{1}{2}\lbrace H[3/2-(q+1/2q)] +H[3/2-(p+1/2p)]\rbrace . \end{aligned}$$

This expression exhibits a contrasting behavior, as compared to the PD, since (51) depends on the probabilities of the players when they use classical strategies. On the other hand, as we can see in Eq. (3), the only strategies that make sense in the classical game are where the probabilities are equal, since for the other strategies, the pay-offs obtained are zero. Therefore, we proceed to evaluate the expression Eq. (51) when $p=q=w$,

52 $$\begin{aligned} \chi ^{(BS)}=\max _{P^{(BS)},Q^{(BS)}} {\mathcal {I}}_{G} -{\mathcal {I}}_{G}\mid {\mathcal {I}}_{\Pi ^{(BS)}}=2- H[3/2-(w+1/2w)]. \end{aligned}$$

The solutions that maximize the information capacity obtained are valid for the probability interval $w \in [1/2 ,1]$. However, for quantum strategies, $w \in [1 / \sqrt{2}, 1]$, so we have

53 $$\begin{aligned} \chi ^{(\star BS)}=\max _{P^{\star (BS)},Q^{\star (BS)}} {\mathcal {I}}_{G} -{\mathcal {I}}_{G}\mid {\mathcal {I}}_{\Pi ^{(BS)}}=2-H[\frac{1}{2}(1-2w)^{2} +2(1-w)\sqrt{w^{2}-1/2}]. \end{aligned}$$

**Figure 1:**
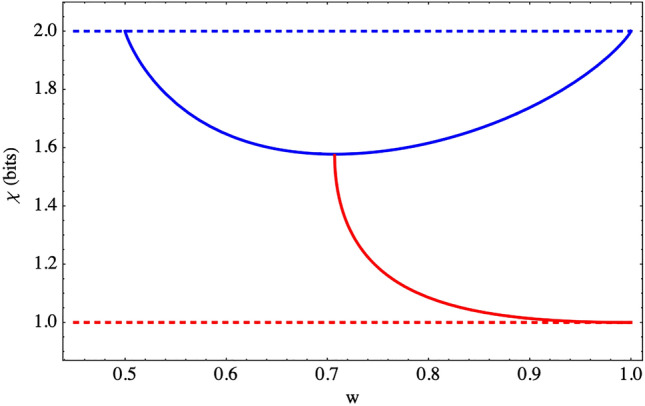
Information Capacity $\chi$ with respect to the probability *w* for the Prisoner’s Dilemma (dashed lines) and Battle of the Sexes (continuous lines) discussed in the text. Depicted in the figure are the classical strategies (online blue) and quantum strategies (online red).

From Eqs. (49) and (50), we see the influence of the term ${\mathcal {I}}_{G}\mid {\mathcal {I}}_{\Pi ^{(PD)}}$, in both information capacities for the PD. The information capacity corresponding to the classical strategies contains more information than the information capacity of the quantum strategies. This is due to the behavior of ${\mathcal {I}}_{G}\mid {\mathcal {I}}_{\Pi ^{(PD)}}$ that satisfy the extreme cases for which the information capacity is maximum and minimum. The values ${\mathcal {I}}_{G}\mid {\mathcal {I}}_{\Pi ^{(PD)}}=-1$ correspond to the classical strategies and ${\mathcal {I}}_{G}\mid {\mathcal {I}}_{\Pi ^{(PD)}}=0$ to the quantum strategies. Thus, we see that the PD reflects the behavior of an ideal channel for classical strategies. On the other hand, for the quantum strategies, the game capacity reached is the lower bound of information possible, due to the presence of entanglement in the game. The entanglement is responsible for increasing the correlation of the game’s input and output information, which is reflected in the term $H(\epsilon _{4}^{\star })$. In particular, for the PD, $H(\epsilon _{4}^{\star (PD)})=1$ achieves the maximum information possible. Therefore, there is a stronger correlation between the game input and output probabilities, as quantified through $H(\epsilon _{4}^{\star })$.

For the expressions in Eqs. (52) and (53), we have the term ${\mathcal {I}}_{G}\mid {\mathcal {I}}_{\Pi ^{(BS)}}>0$, therefore these information capacities are decreasing with the probability *w*, as we see in Fig. 1. That is, we have that for the BS the information capacity depends on the strategies of the players, which is not the case with the PD.

For Eq. (52), we obtain a parabolic behavior, which is similar to that of the Flip Channel^26^. This is due again to the strong symmetry of the game, i.e. the game probabilities $P^{(BS)}$ and $Q^{(BS)}$ are expressed only as a function of the parameter $\epsilon _{1}$, which does not happen for the PD when classical strategies are used. As a consequence of this symmetry, we have that $\chi ^{(BS)}$ depends on the strategies that players take. In this manner, the strategy that maximizes information capacity corresponds to the NE that benefits Bob, whose probability value is $w=1$.

For Eq. (53), we determine a decreasing behavior in the probability *w*, from the sign of ${\mathcal {I}}_{G}\mid {\mathcal {I}}_{\Pi }^{(BS)}$, as discussed above, where we obtained the same sign for both strategies.

In general, we see that the sign change of ${\mathcal {I}}_{G}\mid {\mathcal {I}}_{\Pi }^{\alpha }$ in the games is the main responsible for the decrease or increase in the value of the information capacity. Moreover, only for the BS, the information capacity decreases for both strategies. For this particular game, the entanglement does not drastically affect the information capacity, as happens with the PD, since information values greater than 2 bits are obtained. As with the PD, the entanglement is quantified mainly by the term $H(\epsilon _{4}^{\star BS})$ in determining the game information capacity.

The information capacity in Eq. (53) reaches the maximum of information for the probabilities $w^{(BS)}=1/\sqrt{2}$ ; $u^{(BS)}=0$ or $w^{(BS)}=1/\sqrt{2}$ ; $u^{(BS)}=1$ whose pay-off is $(5/2-\sqrt{2},7/2-2\sqrt{2})$ (see Table 2). This maximum of information occurs around the minimum possible value of information obtained for the classical strategies, as we see in Fig. 1. This is due to two reasons: The first is the symmetry of the game for the terms $\epsilon _{i}^{BS}$. The second one is due to the increase in the correlations of the game input and output information directly related to the presence of entanglement in the quantum game.

### The connection between information and the game strategies, breaking dilemmas

Here we determine the value of the pay-offs for Alice and Bob, first when the joint probabilities minimize and then maximize the game information ${\mathcal {G}}^{\alpha }$, respectively. The game information is minimal for two values of the player information argument, 0 and 1, which we represent as ${\mathcal {G}}^{\alpha }_{0}$ and ${\mathcal {G}}^{\alpha }_{1}$, respectively. When the game information is a maximum, the information capacity of the game is $\chi _{\alpha }$. The quantum strategies are represented by the super-index $\star$. The results obtained are shown in the Table 2.

**Table 2:** Pay-offs when the information is minimal and maximum for Prisoner’s Dilemma and Battle of the Sexes.

${\mathcal {G}}^{(PD)}_{0}$	${\mathcal {G}}^{(PD)}_{1}$	${\mathcal {G}}^{\star (PD)}_{0}$	${\mathcal {G}}^{\star (PD)}_{1}$	$\chi ^{(PD)}$	$\chi ^{\star (PD)}$
$\Pi _{A}^{(1)}=1 \quad \Pi _{A}^{(2)}=1-p$	$\Pi _{A}=3$	$\Pi _{A}^{(1=2)}=1$	$\Pi _{A}^{(1=2)}=3$	$\Pi _{A}=3/2$	$\Pi _{B}^{(1=2)}=2$
$\Pi _{B}^{(1)}=1 \quad \Pi _{B}^{(2)}=4p+1$	$\Pi _{B}=3$	$\Pi _{B}^{(1=2)}=1$	$\Pi _{B}^{(1=2)}=3$	$\Pi _{B}=4$	$\Pi _{B}^{(1=2)}=2$

${\mathcal {G}}^{(BS)}_{0}$	${\mathcal {G}}^{(BS)}_{1}$	${\mathcal {G}}^{\star (BS)}_{0}$	${\mathcal {G}}^{\star (BS)}_{1}$	$\chi ^{(BS)}$	$\chi ^{\star (BS)}$
$\Pi _{A}^{(1)}=1-q$	$\Pi _{A}=2$	$\Pi _{A}^{(1=2)}=1$	$\Pi _{A}^{(1=2)}=2$	$\Pi _{A}^{(1=2)}\vert _{q=1/2 ;\ p=1}=1$	$\Pi _{A}^{(1=2)}\vert _{w=1/\sqrt{2}}=\frac{5}{2}-\sqrt{2}$
$\Pi _{A}^{(2)}=1-p$				$\Pi _{A}^{(1=2)}\vert _{p=1/2 ;\ q=1}=1$	$\Pi _{A}^{(1=2)}\vert _{w=1}=3/2$
$\Pi _{B}^{(1)}=2(1-q)$	$\Pi _{B}=1$	$\Pi _{B}^{(1=2)}=2$	$\Pi _{B}^{(1=2)}=1$	$\Pi _{B}^{(1=2)}\vert _{q=1/2 ;\ p=1}=1/2$	$\Pi _{B}^{(1=2)}\vert _{w=1/\sqrt{2}}=\frac{7}{2}-2\sqrt{2}$
$\Pi _{B}^{(2)}=2(1-p)$				$\Pi _{B}^{(1=2)}\vert _{p=1/2 ;\ q=1}=1/2$	$\Pi _{B}^{(1=2)}\vert _{w=1}=3/2$

In particular for quantum strategies, we obtain the NE where the game dilemma is lifted for strategies $w^{(PD)}=1$ ; $u^{(PD)}=0$ or $w^{(PD)}=1$ ; $u^{(PD)}=1$. The above strategies are the same, these correspond to the quantum strategy $({\mathcal {Q}}, {\mathcal {Q}})=(3,3)$ obtained by^6,7^. As in Table 1, we obtained results that show the advantages of the entanglement in overcoming the games dilemma for the classical and quantum strategies. In Table 2, we obtain the pay-offs that maximize the game information, that is, the pay-offs corresponding to the information capacity. From the latter we obtain, for the classic strategies, advantageous pay-offs for Bob over Alice, in contrast to the quantum strategies where we obtain an equilibrium.

In Table 2 we obtain the NE for classical and quantum strategies. The pay-offs corresponding to the information capacity are $\chi ^{(BS)}$ and $\chi ^{\star (BS)}$. For the classical strategies, there are advantageous pay-offs for Alice and Bob. However, with quantum strategies, we get non-trivial results; for the games (see Table 2), we get one equilibrium and a strategy whose payoffs are beneficial to the players. In particular, this strategy for the BS we get the pay-off $(5/2-\sqrt{2},7/2-2\sqrt{2})$ for the strategy $w^{(BS)}=1/\sqrt{2}$, which benefits Alice. It is important to point out that for the BS in Table 1, a NE is obtained due to the presence of the entanglement in the game, which did not solve the dilemma for the game as it happens with Eisert quantum games^6,7^. However, with the incorporation of information in QG from joint probabilities, we have obtained a game equilibrium that solves the original dilemma of the games whose pay-off is (3/2, 3/2) for the probabilities $w^{(BS)}=1$ ; $u^{(BS)}=\frac{1}{2}(1-\frac{1}{\sqrt{2}})$ or $w^{(BS)}=1$ ; $u^{(BS)}=\frac{1}{2}(1+\frac{1}{\sqrt{2}})$.

The aforementioned equilibrium was found by^14^, inspired by the game approach of^9^, when using maximally entangled states. In our game approach, we have solved the dilemma of the two games through the same formalism due to the presence of entanglement in the game, either through the approach of the joint probabilities or through the information in the games, specifically through the game information capacity.

The incorporation of the information shows the real advantages of entanglement in the games, contrary to what happens with the NE. Therefore, we show a new way of breaking the dilemma: Determine the value of the pay-offs for the players when the joint probabilities maximize or minimize the game information. Finally, we see the full manifestation of entanglement in QG, since it is maximum for our game approach, we see how the incorporation of the proposed definition of the information capacity (48) to the game strategies offers advantages to the players such as reaching new equilibria that allow for overcoming the classical dilemma in the classical games.

## Summary and conclusions

We have shown a description of QG in terms of joint probabilities through the formalism proposed in reference^22^. From the entanglement probability ($\epsilon _{1}^{\star }$), which quantifies the joint probability correlations, we obtained that QG are characterized by non-factorizable joint probabilities, due to entanglement. The approach taken to determine non-factorizability of the entanglement probability is associated with the probabilities and the information introduced by quantum strategies. Furthermore, we determined the inequalities associated with the Nash Equilibrium (NE) for two emblematic games: The Prisoner’s Dilemma and The Battle of the Sexes. The NE condition allows for the dissolution of the game’s dilemma for the Prisoner’s Dilemma, through the strategies associated with the NE (see Table 1). In contrast, for the Battle of the Sexes, the dilemma is not lifted, due to the symmetry of the game. The latter shows that the NE condition is not enough to dissolve the dilemma in all games through entanglement, as obtained for the Eisert’s QG^6,7^. However, when we introduce the *game information* i.e. determine the value of the pay-offs for the players when the joint probabilities either maximizes or minimizes the game information for the QG, (what we refer to as *the limit values of the game information*), we break the dilemma for all games. In particular, we obtained an equilibrium for the Battle of the Sexes (see Table 2) that breaks the dilemma and does not correspond to the NE and thus find a new route to cooperation between players.

In expressing QG in terms of joint probabilities, we propose a way to measure information for the game understanding it as a channel. We have also defined the Information Capacity of a Quantum Game through the Joint Probabilities. For the Prisoner’s Dilemma, when players apply classical strategies, we obtain that the information in the game is superior to when they take quantum strategies. This is due to the correlations of the joint probabilities generated by entanglement. The entanglement directly affects the game information, through the term ${\mathcal {I}}_{G}\mid {\mathcal {I}}_{\Pi ^{\alpha }}$ in Eq. (46). On the other hand, the information capacity for the Battle of the Sexes has maxima and minima, which are associated with the strategies of the players. In the latter game, the information is a maximum when the players use strategies associated with the Nash Equilibrium. Here, we saw how symmetry is reflected in the probabilities $\epsilon _{i}^{\alpha }$ (see Eqs. (21), (34) and (35)) and the entanglement influence on the behavior of the information capacity.

This definition of information offers an advantage over other games approaches^5–7,9^, since the game information is maximized from the probabilities of the game. Consequently, we can build new games from the parameters $\epsilon _{i}^{\alpha }$, for the joint probabilities, which allows for a better maximization of information.

When assessing the behavior of the information capacity for Battle of the Sexes in Fig. 1, we obtain better performance than with the Flip Channel^26^, since the information capacity for the classical strategy does not decay to 1 bit^26^. Thus, we can compare Szopa’s efficiency with the better performance obtained for the information capacity above, since the incorporation of the Fra̧ckiewicz-Pykacz Parameterization^32^, expands the possibilities for finding equilibria that would not usually be found in classical games. This introduces correlations, which is equivalent to our game approach by introducing Non-factorizable probabilities for QG in the Eisert’s approach. Likewise, we see better performance with the quantum and classical strategies with respect to the Flip Channel, for the Prisoner’s Dilemma by using quantum and classical strategies. Particularly the quantum strategy has better performance for $w > 1/2$ than the usual noisy channels in Quantum information theory^1,26^. In this sense, we can conjecture that the game formalism proposed by incorporating noise could offer a better performance in the transmission of information in quantum channels for the quantum or classical strategies.

Furthermore, the equilibria obtained for the games, in our formalism, can expect to be maintained when incorporating noise, as occurs with the NE for the Prisoner’s Dilemma when incorporating decoherence^36^.

Regarding applications beyond the scope of quantum games, we have discussed considering quantum games as information channels with a channel capacity. The channel capacity is related to its tolerance to noise and decoherence. We have shown that the game strategies breaking dilemmas in the BS has a higher capacity than the flip channel and thus our new approach might be thought to of a new route to high capacity information channel resilient to noise.

Another application mentioned in the introduction relates to mapping of game concepts onto statistical mechanics describing thermalization processes using classical game concepts^31^. Exploring the quantum game counterpart could be illuminating for identifying decoherence procesess understood through quantum game equilibria as described here. Finally, for some time there have been attempts at mapping and many body physics, where one may think that energy eigenstates of a quantum many-body Hamiltonian can be considered as strategies adopted by the quantum particles to attain a configuration that satisfies the energy constraints. The payoffs can be mapped onto total energy constraints and the ground state a manifestation of the best strategy of the game^37^. The straightforward way in which the joint probabilities identify the best strategies may then serve as a way to compute the ground state of a many body system.

## Data Availability

All data generated or analysed during this study are included in this published article.

## References

[1] Nielsen MA, Chuang IL (2011). Quantum computation and quantum information.

[2] Khan FS, Solmeyer N, Balu R (2018). Quantum games: a review of the history, current state, and interpretation. Quantum Inf. Proc..

[3] Flitney AP, Abbott D (2002). An introduction to quantum game theory. Fluct. Noise Lett..

[4] Iqbal, A. *Studies in the Theory of Quantum Games*, arXiv:quant-ph/0503176.

[5] Meyer DA (1999). Quantum strategies. Phys. Rev. Lett..

[6] Eisert J, Wilkens M, Lewenstein M (1999). Quantum games and quantum strategies. Phys. Rev. Lett..

[7] Eisert J, Wilkens M (2000). Quantum games. J. Mod. Opt..

[8] Benjamin SC, Hayden PM (2001). Comment on “Quantum Games and Quantum Strategies”. Phys. Rev. Lett..

[9] Marinatto L, Weber T (2000). A quantum approach to static games of complete information. Phys. Lett. A..

[10] Ikeda K, Aoki S (2022). Theory of quantum games and quantum economic behavior. Quantum Inf. Proc..

[11] Ikeda K (2021). Quantum contracts between Schrödinger and a cat. Quantum Inf. Proc..

[12] Frackiewicz P (2018). Quantum approach to Cournot-type competition. Int. J. Theor. Phys..

[13] Enk SJ, Pike R (2002). Classical rules in quantum games. Phys. Rev. A..

[14] Nawaz A, Toor AH (2004). Dilemma and quantum battle of sexes. J. Phys. A Math. Gen..

[15] Benjamin SC, Hayden PM (2001). Multiplayer quantum games. Phys. Rev. A.

[16] Du J, Li H, Xu X, Zhou X, Han R (2002). Entanglement enhanced multiplayer quantum games. Phys. Lett. A.

[17] Du J, Li H, Xu X, Zhou X, Han R (2002). Multi-player and multi-choice quantum game. Chin. Phys. Lett..

[18] Flitney AP, Abbott D (2004). Quantum two and three person duels. J. Opt. B Quantum Semiclass Opt..

[19] Li Q, He Y, Jiang J (2009). A novel clustering algorithm based on quantum games. J. Phys. A Math. Gen..

[20] Iqbal A, Cheon T (2007). Constructing quantum games from nonfactorizable joint probabilities. Phys. Rev. E..

[21] Chappell JM, Iqbal A, Abbott D (2010). Constructing quantum games from symmetric non-factorizable joint probabilities. Phys. Rev. E..

[22] Iqbal A, Chappell JM, Abbott D (2016). On the equivalence between non-factorizable mixed-strategy classical games and quantum games. R. Soc. Open Sci..

[23] Jiménez E (2003). Quantum games: mixed strategy Nash’s equilibrium represents minimun entropy. Entropy..

[24] Hidalgo EG (2007). Quantum games entropy. Phys. A.

[25] Kak S (2016). State ensembles and quantum entropy. Int. J. Theor. Phys..

[26] Liang X, Fan H (2002). Entanglement-assisted classical capacities of some single qubit quantum noisy channels. Mod. Phys. Lett. B..

[27] Ikeda K, Aoki S (2021). Infinitely repeated quantum games and strategic efficiency. Quantum Inf. Proc..

[28] Plenio MB, Virmani S (2007). An introduction to entanglement measures. Quant. Inf. Comp..

[29] Horodecki M (2007). Entanglement Measures. Quant. Inf. Comp..

[30] Bru D (2002). Characterizing entanglement. J. Math. Phys..

[31] Babajanyan SG, Allahverdyan AE, Cheong KH (2020). Energy and entropy: Path from game theory to statistical mechanics. Phys. Rev. Res..

[32] Szopa M (2021). Efficiency of classical and quantum games equilibria. Entropy.

[33] Maschler M, Solan S, Zamir S (2020). Game theory.

[34] Rasmusen E (2005). Games and information: An introduction to game theory.

[35] Du, J., Xu, X., Li, H., Zhou, X., & Han, R. *Nash Equilibrium in the Quantum Battle of Sexes Game*, arXiv:quant-ph/0010050.

[36] Chen LK, Ang H, Kiang D, Kwek LC, Lo CF (2003). Quantum prisoner dilemma under decoherence. Phys. Lett. A.

[37] Roy SS, Bera A, Sierra G (2022). Simulating violation of causality using a topological phase transition. Phys. Rev. A.

